# [Corrigendum] miR-221/222 promote malignant progression of glioma through activation of the Akt pathway

**DOI:** 10.3892/ijo.2026.5937

**Published:** 2026-09-03

**Authors:** Junxia Zhang, Lei Han, Youlin Ge, Xuan Zhou, Anling Zhang, Chunzhi Zhang, Yue Zhong, Yongping You, Peiyu Pu, Chunsheng Kang

Int J Oncol 36: 913-920, 2010; DOI: 10.3892/ijo_00000570

Following the publication of the above paper, and an Expression of concern statement (doi: 10.3892/ijo.2025.5808) that was published to draw the readers' attention to the fact that, for the immunohistochemistry images shown in Fig. 6, the 'Control/PCNA' and 'Control/p27kip1' panels appeared to be duplicates of each other, the authors have now responded concerning this issue. After having examined their original data, the authors have realized that this figure was inadvertently assembled incorrectly; specifically, the Control/p27kip1 panel, as shown correctly, was duplicated in the Figure as the Control/PCNA panel. The revised version of Fig. 6, now showing the correct data for the Control/PCNA panel, is shown on the next page. Note that this error did not affect the overall conclusions reported in the study. The authors are grateful to the Editor of *International Journal of Oncology* for allowing them this opportunity to publish a Corrigendum, and all the authors agree with its publication; furthermore, the authors apologize to the readership for any inconvenience caused.

## Figures and Tables

**Figure 6:**
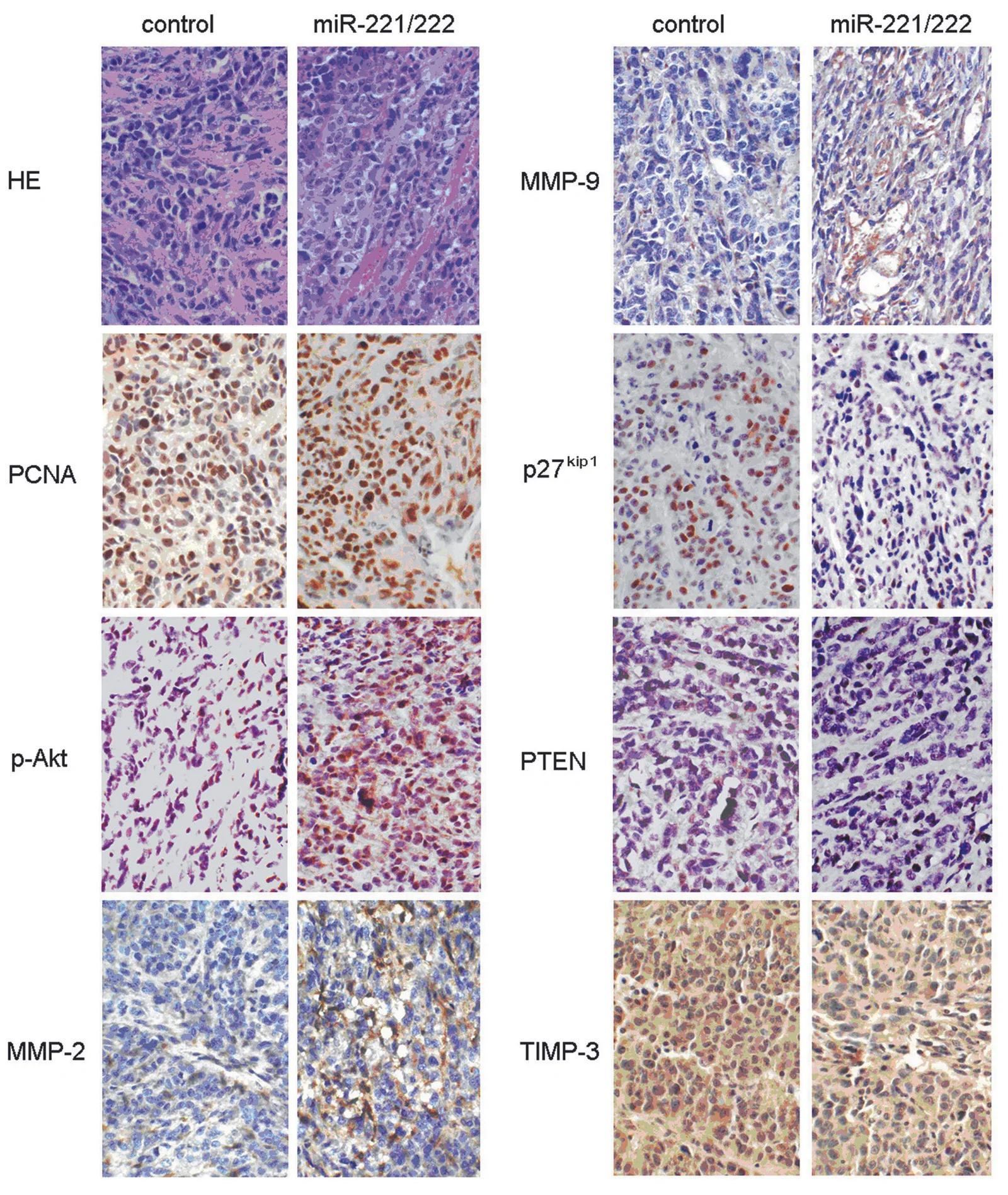
Immunohistochemistry analysis of xenograft tumors after miR-221/222 treatment. The expression levels of PCNA, p-Akt, MMP-2 and MMP-9 were increased in U251 xenograft tumors of miR-221/222 group. Additionally, the expression levels of p27kip1, PTEN and TIMP-3 were suppressed

